# Metformin Enhances the Pro‐Regenerative Function of Human Adipose‐Derived Stromal Cells for Diabetic Wound Healing

**DOI:** 10.1111/wrr.70214

**Published:** 2026-10-06

**Authors:** Kristina Lee Khaw, Max von Suskil, Andrew Bohner, Russell J. Buono, Steven C. Bonawitz, Kathryn Behling, Michael E. DiSanto, Ping Zhang

**Affiliations:** ^1^ Cooper Medical School of Rowan University Camden New Jersey USA; ^2^ Cooper University Health Care Camden New Jersey USA

**Keywords:** adipose‐derived stromal cells, angiogenesis, biomaterials, metformin, wound healing hyperglycaemia

## Abstract

Chronic wounds associated with diabetes remain a major challenge due to impaired angiogenesis, reduced fibroblast activity and dysfunction of regenerative cells. Adipose‐derived mesenchymal stromal cells (ASCs) represent a promising therapeutic option; however, their regenerative potential is limited under hyperglycaemic conditions. This study investigated whether metformin could enhance ASC function and improve their therapeutic potential for tissue regeneration. Transcriptomic analysis of wound tissue identified pathways associated with angiogenesis, inflammation, extracellular matrix remodelling and cell survival, including PI3K–AKT, JAK–STAT and AGE–RAGE signalling. Functionally, metformin increased ASC proliferation in a concentration‐dependent manner and promoted sustained cell expansion over time. Metformin‐treated ASCs also enhanced fibroblast migration in a co‐culture system, indicating improved paracrine pro‐reparative signalling. Metformin further increased the secretion of angiogenic factors, including vascular endothelial growth factor (VEGF) and fibroblast growth factor (FGF), supporting a pro‐angiogenic secretory profile. Metformin enhanced CD31 expression and tube formation capacity of ASCs under normoglycemic conditions, particularly in combination with VEGF; however, these effects were attenuated under high‐glucose conditions. Additionally, metformin improved ASC adhesion to small intestinal submucosa (SIS) scaffolds and supported endothelial marker expression in a three‐dimensional biomaterial environment. Collectively, these findings demonstrate that metformin promotes a pro‐regenerative ASC phenotype characterized by enhanced proliferation, paracrine signalling, endothelial‐associated responses and scaffold integration. However, hyperglycaemic conditions partially impair these effects, highlighting the influence of metabolic stress on regenerative responses. Overall, metformin preconditioning represents a promising strategy to enhance ASC‐based therapies for chronic and diabetic wound healing.

## 1 Introduction

Diabetes mellitus (DM) is a chronic metabolic disease projected to affect 592 million people worldwide by 2035 [1]. In the United States, approximately 31.3 million individuals were living with diabetes in 2019 and 15%–25% will develop diabetic foot ulcers (DFUs) during their lifetime [2, 3]. Both Types 1 and 2 diabetes are associated with an increased risk of DFUs, with incidence rates approaching 25% [4, 5]. Nearly half of all DFUs occur in patients with peripheral artery disease (PAD), further compromising tissue perfusion and wound healing. Older adults are at particularly high risk for DFU‐related complications, including infection, abscess formation and osteomyelitis [6, 7]. As the prevalence of diabetes continues to rise, chronic non‐healing wounds represent an increasing clinical and socioeconomic burden.

Cell‐based therapies have emerged as promising approaches for enhancing diabetic wound repair. Transplantation of endothelial progenitor cells (EPCs) and mesenchymal stromal cells (MSCs) has been shown to accelerate healing in experimental diabetic wound models [8, 9]. MSCs derived from bone marrow, peripheral blood, umbilical cord and adipose tissue promote tissue regeneration through angiogenesis, immunomodulation, re‐epithelialization and extracellular matrix (ECM) remodelling [10, 11]. Among these cell sources, human adipose‐derived mesenchymal stromal cells (ASCs) are particularly attractive because of their abundance, ease of isolation and potent pro‐angiogenic and immunomodulatory properties [12, 13, 14, 15]. ASCs contribute to wound healing through the secretion of growth factors, cytokines and extracellular vesicles (EVs) that stimulate fibroblast migration, proliferation and collagen synthesis, as well as through their capacity to acquire endothelial‐like characteristics under appropriate conditions [12, 13, 16]. However, direct cell injection often results in poor cell retention and limited structural support at the wound site [17, 18]. Biomaterial scaffolds have therefore been developed to improve cell survival, adhesion and engraftment, thereby enhancing therapeutic efficacy [19, 20, 21].

Metformin (MTF), the first‐line pharmacological treatment for Type 2 diabetes, has recently attracted interest in regenerative medicine because of its cytoprotective and pro‐regenerative properties. MTF activates AMP‐activated protein kinase (AMPK), promotes mitochondrial homeostasis, reduces oxidative stress and has been associated with activation of angiogenic signalling pathways, including PI3K/Akt, eNOS and Notch signalling [22, 23, 24]. In parallel, MTF‐loaded biomaterials have been developed as localized delivery systems to improve diabetic wound healing outcomes [25, 26]. Despite these advances, it remains unclear whether MTF can enhance the regenerative and angiogenic potential of ASCs, particularly, within a scaffold‐based microenvironment under hyperglycaemic conditions.

Therefore, the objective of this study was to investigate the effects of MTF on ASC proliferation, paracrine activity, endothelial differentiation and biomaterial interactions under normoglycemic and hyperglycaemic conditions. Using small intestinal submucosa (SIS) scaffolds as a clinically relevant biomaterial platform, we evaluated ASC adhesion, endothelial marker expression, angiogenic function and fibroblast migration in response to MTF treatment. In addition, RNA sequencing was performed to identify molecular pathways associated with the observed functional changes. We hypothesized that MTF acts as a metabolic modulator that enhances ASC regenerative function and promotes a pro‐regenerative phenotype, with potential benefits under hyperglycaemic conditions. Together, these studies provide mechanistic and translational insights into the use of MTF‐preconditioned ASCs for scaffold‐based therapies aimed at improving diabetic wound healing.

## 2 Materials and Methods

### 2.1 Cell Culturing

Human adipose‐derived stromal cells (ASCs) were obtained from the American Type Culture Collection (ATCC, Manassas, VA, USA) and cultured according to the manufacturer's instructions. Cells were initially expanded in mesenchymal stem cell basal medium supplemented with a growth kit (ATCC) and subsequently maintained in M199 medium supplemented with 10% foetal bovine serum (FBS) and antibiotics/antimycotics (Thermo Fisher Scientific, Waltham, MA, USA). The culture medium was changed twice weekly and cells were passaged at approximately 80% confluence using a 1:3 split ratio. Cells between Passages 2 and 5 were used for all experiments.

Human primary dermal fibroblasts (HDFs) were also obtained from ATCC and cultured in basal medium supplemented with the appropriate growth kit according to the manufacturer's protocol.

### 2.2 MTF Treatment and Cell Viability Assays

MTF (Sigma‐Aldrich, St. Louis, MO, USA) was dissolved in sterile water to prepare stock solutions and stored at −20°C. Cell viability was assessed using the MTT assay. ASCs were seeded at a density of 1 × 10^4^ cells per well in 24‐well plates and treated with MTF at concentrations of 0.1, 1 or 10 mM for 24 or 48 h. The selected concentration range (0.1–10 mM) was based on preliminary viability testing and previously published studies demonstrating its effectiveness and non‐cytotoxic effects in MSCs and endothelial cells.

For the MTT assay, 500 μL of MTT solution (5 mg/mL; Sigma‐Aldrich) was added to each well and incubated for 3 h at 37°C in a humidified atmosphere containing 5% CO_2_. After incubation, the medium was removed and formazan crystals were dissolved in 200 μL of DMSO. Absorbance was measured at 570 nm using a microplate reader. Untreated cells served as the control group. All experiments were performed in triplicate.

### 2.3 Cell Proliferation and Doubling Time

Cell proliferation was evaluated by constructing growth curves over 8 days. ASCs were plated at a density of 5 × 10^3^ cells/cm^2^ in M199 medium and treated with 0.1 mM MTF, with or without 25 mM glucose. The culture medium was replenished every 3 days. At specified time points, cells were trypsinized and counted using a Countess II Cell Counter (Thermo Fisher Scientific).

Doubling time (DT) during the logarithmic growth phase was calculated using the following formula: DT = (*t* − *t*_0_) × log2/(log*N* − log*N*_0_), where *t* and *t*_0_ represent time points and *N* and *N*_0_ represent the corresponding cell counts.

### 2.4 Enzyme‐Linked Immunosorbent Assay (ELISA)

The concentrations of vascular endothelial growth factor (VEGF) and basic fibroblast growth factor (bFGF) in cell culture supernatants were measured using quantitative sandwich ELISA kits (Thermo Fisher Scientific) according to the manufacturer's instructions. ASCs were cultured with 0.1 mM MTF, with or without 25 mM glucose, for 72 h. Culture supernatants were collected and centrifuged to remove cellular debris prior to analysis. Briefly, samples and standards were added to 96‐well plates pre‐coated with capture antibodies specific for human VEGF or bFGF, followed by incubation with detection antibodies and subsequent addition of the enzyme‐linked reagent. Colorimetric development was measured at 450 nm using a microplate reader.

### 2.5 Three‐Dimensional (3D)‐Printed Insert–Based Wound Scratch Assay and Transwell Co‐Culture System

Cell migration was evaluated using a modified wound‐healing assay combined with a Transwell co‐culture system incorporating a 3D‐printed insert. Human dermal fibroblasts (HDFs) were seeded in 24‐well plates at a density of 1 × 10^5^ cells per well and allowed to reach confluence. A sterile 3D‐printed insert was then used to generate a uniform scratch wound. Cells were incubated for 24 h to allow attachment and stabilization of the wound area. Separately, human ASCs were cultured in 24‐well Transwell inserts (Thermo Fisher Scientific) and treated with either 0.1 mM MTF or vehicle control in the presence or absence of 25 mM glucose (high glucose condition). After fibroblast attachment, the inserts were removed and baseline (0 h) images of the wound area were acquired. The ASC‐containing inserts were then transferred onto the fibroblast culture plates to establish the co‐culture system and fresh medium was added. After 24 h of co‐culture, images were captured again (24 h). Wound closure was quantified using ImageJ software and the relative migration rate was calculated as (wound area at 0 h − wound area at 24 h)/wound area at 0 h × 100%. All experiments were performed independently in triplicate.

### 2.6 Endothelial Differentiation of ASCs

To induce endothelial differentiation, ASCs were cultured in M199 medium supplemented with 50 ng/mL VEGF, with or without MTF, for up to 10 days. ASCs cultured in M199 medium alone served as controls. Endothelial differentiation was evaluated by assessing endothelial cell–specific markers using quantitative real‐time PCR (qPCR), immunofluorescence (IF) staining and Matrigel‐based tube formation assays.

### 2.7 qPCR

Total RNA was extracted using TRIzol reagent (Thermo Fisher Scientific) according to the manufacturer's instructions. Complementary DNA (cDNA) was synthesized from total RNA using a reverse transcription kit. Gene expression was analysed using specific primers for platelet endothelial cell adhesion molecule (CD31) (Forward: 5′‐CACAGCAATTCCTCAGGCTA‐3′; Reverse: 5′‐TTCAGCCTTCAGCATGGTAG‐3′) and VEGF (Forward: 5′‐ATCACGAAGTGGTGAAGTTC‐3′; Reverse: 5′‐TGCTGTAGGAAGCTCATCTC‐3′). GAPDH was used as an internal control (Forward: 5′‐GGATCTCGCTCCTGGAAGATG‐3′; Reverse: 5′‐GCACCGTCAAGGCTGAGAAC‐3′). qPCR reactions were performed using standard conditions and relative gene expression was calculated using the 2−^ΔΔ*Ct*^ method.

### 2.8 Tube Formation Assay

The angiogenic potential of ASCs was evaluated using a Matrigel‐based tube formation assay. After 10 days of endothelial differentiation in medium with or without MTF, ASCs were seeded onto growth factor–reduced Matrigel (2 × 10^5^ cells per 100 μL). Cells were incubated at 37°C in a humidified atmosphere containing 5% CO_2_ for 6–12 h and the formation of tube‐like structures was observed using phase‐contrast microscopy. Angiogenic capacity was quantified by measuring total tube length in five randomly selected fields per group. ASCs cultured in M199 medium without differentiation factors served as undifferentiated controls.

### 2.9 Scaffold Adhesion and Endothelial Differentiation on SIS Scaffold

Human ASCs were resuspended in culture medium with or without MTF at a density of 2 × 10^6^ cells/mL. To evaluate scaffold adhesion, 0.5 mL of cell suspension (1 × 10^6^ cells) was seeded onto SIS sheets (1.5 cm^2^; Cook Biotech, IN, USA), which served as a soft tissue graft model. After 4 h of incubation to allow initial attachment, additional culture medium was added and cells were maintained for 3 days.

Cell attachment and morphology on the SIS scaffold were assessed by CellTracker Green staining (Thermo Fisher Scientific). To evaluate endothelial differentiation on the scaffold, ASCs cultured on SIS were further maintained in endothelial induction medium supplemented with VEGF, with or without MTF, for 10 days. Endothelial differentiation was assessed by IF staining for the endothelial marker CD31 (Invitrogen, CA, USA) and visualized using a Nikon A1 laser scanning confocal microscope.

### 2.10 IF

ASCs–SIS constructs were fixed in 4% paraformaldehyde for 20 min at room temperature and blocked with 5% bovine serum albumin (BSA) in PBS for 1 h. Samples were then incubated overnight at 4°C with a primary antibody against CD31 (1:100, Abcam). After washing, samples were incubated with a species‐specific fluorescent secondary antibody. Nuclei were counterstained with DAPI using VECTASHIELD mounting medium (Vector Laboratories, CA, USA). Fluorescent images were acquired using a Nikon A1 laser scanning confocal microscope. CD31‐positive cells were quantified using NIH ImageJ software by counting five randomly selected fields per sample and the average number of positive cells per field was calculated.

### 2.11 RNA‐Sequencing

Total RNA was extracted from ASCs cultured under control conditions, treated with MTF, exposed to high glucose or treated with MTF under high‐glucose conditions using TRIzol reagent (Thermo Fisher Scientific) according to the manufacturer's instructions. A total of 12 samples were analysed, with three independent biological replicates per experimental group. RNA sequencing library preparation, sequencing and initial data processing were performed by Novogene US Subsidiary and the UC Davis Genome Center (Sacramento, CA, USA).

Differentially expressed genes (DEGs) were identified using a cutoff of |log2 fold change| ≥ 1.5 and a Bonferroni‐adjusted *p*‐value ≤ 0.05. Heatmaps were generated to visualize gene expression patterns across experimental groups. Functional annotation and enrichment analysis of DEGs were performed using Gene Ontology (GO), including biological process, cellular component and molecular function categories. Pathway enrichment analysis was performed using the Kyoto Encyclopaedia of Genes and Genomes (KEGG) database. Statistical significance for enrichment analyses was determined using the criteria specified for the corresponding analyses [27, 28, 29].

### 2.12 Statistical Analysis

Data are presented as mean ± standard deviation (SD). The number of independent biological experiments (*n*) is indicated in the corresponding figure legends. Technical replicates were averaged to obtain a single value for each biological replicate before statistical analysis. Comparisons between two groups were performed using an unpaired two‐tailed Student's *t*‐test. Comparisons among multiple groups were performed using one‐way or two‐way analysis of variance (ANOVA), as appropriate, followed by the appropriate post hoc multiple‐comparison test. A *p* value < 0.05 was considered statistically significant.

## 3 Results

### 3.1 MTF Modulates Gene Expression and Signalling Pathways in ASCs

To characterize MTF‐induced molecular changes in adipose‐derived stem cells (ASCs), RNA sequencing was performed under normal‐ and high‐glucose conditions. Hierarchical clustering based on global gene expression profiles demonstrated distinct transcriptional patterns among the four experimental groups (MTF + HG, MTF, COL and HG), indicating that both MTF treatment and glucose conditions substantially influenced gene expression in ASCs (Figure 1A). Differential expression analysis revealed extensive transcriptional reprogramming following MTF treatment. Compared with corresponding untreated controls (COL) under matched glucose conditions, MTF treatment resulted in 1613 significantly upregulated genes and 1543 significantly downregulated genes (Figure 1B). Several of the most significantly altered genes included CSF3, ACPP, CAMK2B, PRR9 and TSGA10IP among the upregulated transcripts, whereas IL21‐AS1 was among the most significantly downregulated genes.

**FIGURE 1:**
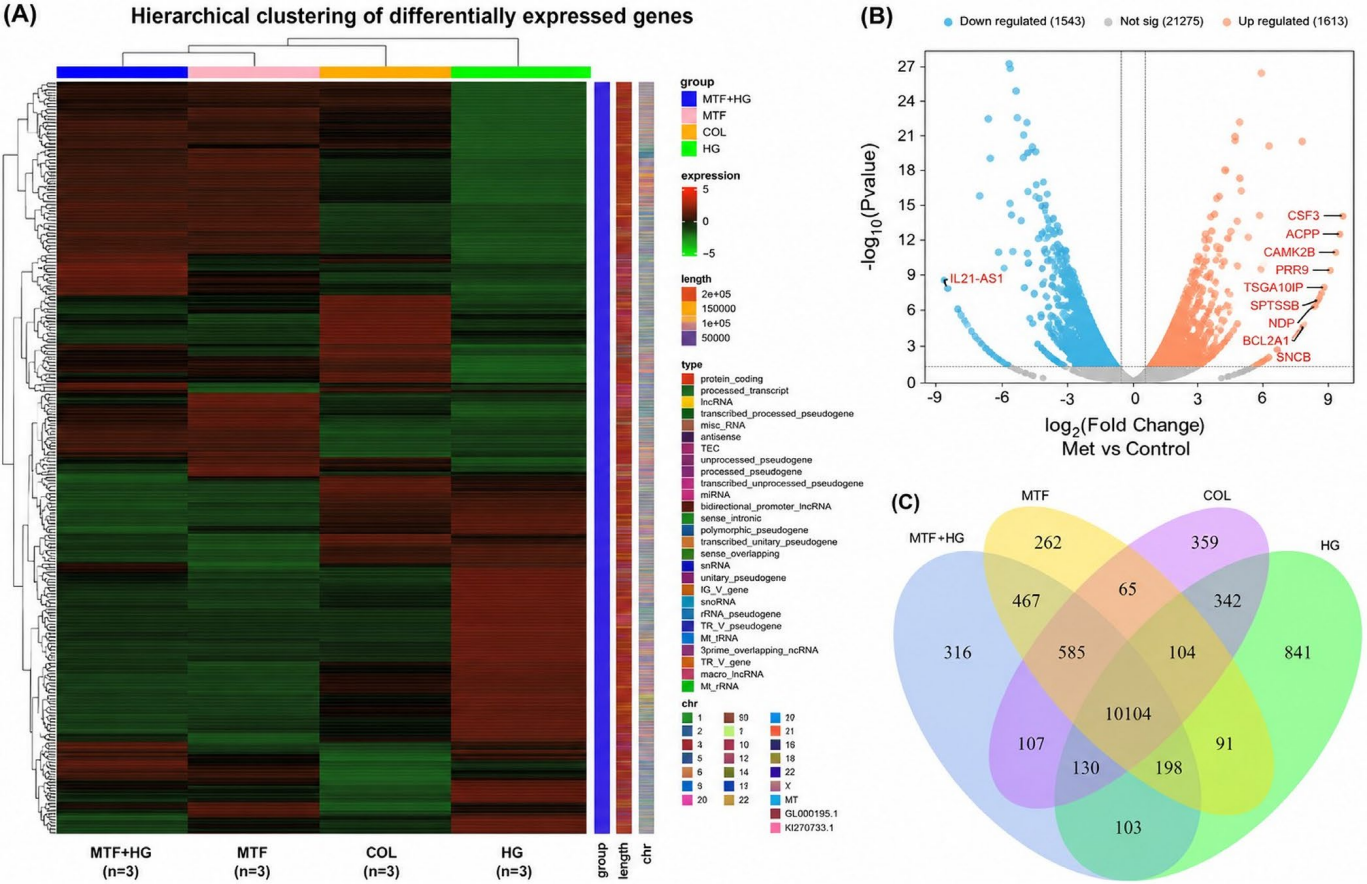
RNA sequencing reveals metformin‐induced transcriptional reprogramming in ASCs under normal and high‐glucose conditions. (A) Hierarchical clustering heatmap showing global gene expression patterns of adipose‐derived stromal cells (ASCs) cultured under control (COL), metformin‐treated (MTF), high‐glucose (HG) and metformin‐treated high‐glucose (MTF + HG) conditions. Each group contains three biological replicates (*n* = 3). Red and green represent relatively increased and decreased gene expression levels, respectively. (B) Volcano plots showing differentially expressed genes (DEGs) identified by comparison of metformin‐treated and untreated ASCs under normal‐glucose conditions (MTF vs. COL) and high‐glucose conditions (MTF + HG vs. HG). Significantly upregulated genes are indicated in orange, significantly downregulated genes in blue and non‐significant genes in grey. Selected representative DEGs are labelled. Differential expression analysis was performed using the predefined criteria of |log_2_ fold change| ≥ 1.5 and adjusted *p* ≤ 0.05. (C) Venn diagram showing overlapping and unique DEG sets among the experimental groups. The analysis demonstrates shared and condition‐specific transcriptional responses associated with metformin treatment under normal‐ and high‐glucose conditions.

To further assess the overlap and specificity of transcriptional responses across treatment conditions, Venn diagram analysis was performed. A substantial proportion of genes were shared among treatment conditions, indicating common transcriptional responses, whereas distinct subsets of genes were uniquely associated with individual groups. Notably, 316 genes were uniquely expressed in the MTF + HG group and 107 genes were uniquely expressed in the MTF group, suggesting that MTF induces both conserved and glucose‐dependent transcriptional programmes in ASCs (Figure 1C).

### 3.2 MTF Enhances ASC Proliferation and Paracrine Secretion of Angiogenic Factors

Based on transcriptomic evidence indicating enrichment of angiogenesis‐related pathways, we next investigated whether MTF influences ASC proliferation and paracrine function.

MTT assays demonstrated that MTF enhanced ASC metabolic activity at low concentrations. After 48 h, 0.1 and 1 mM MTF significantly increased metabolic activity, whereas higher concentrations (5 and 10 mM) had no significant effect (Figure 2A, *p* < 0.05). Based on these findings and the proliferative effects observed at low concentrations, 0.1 mM MTF was selected for subsequent experiments.

**FIGURE 2:**
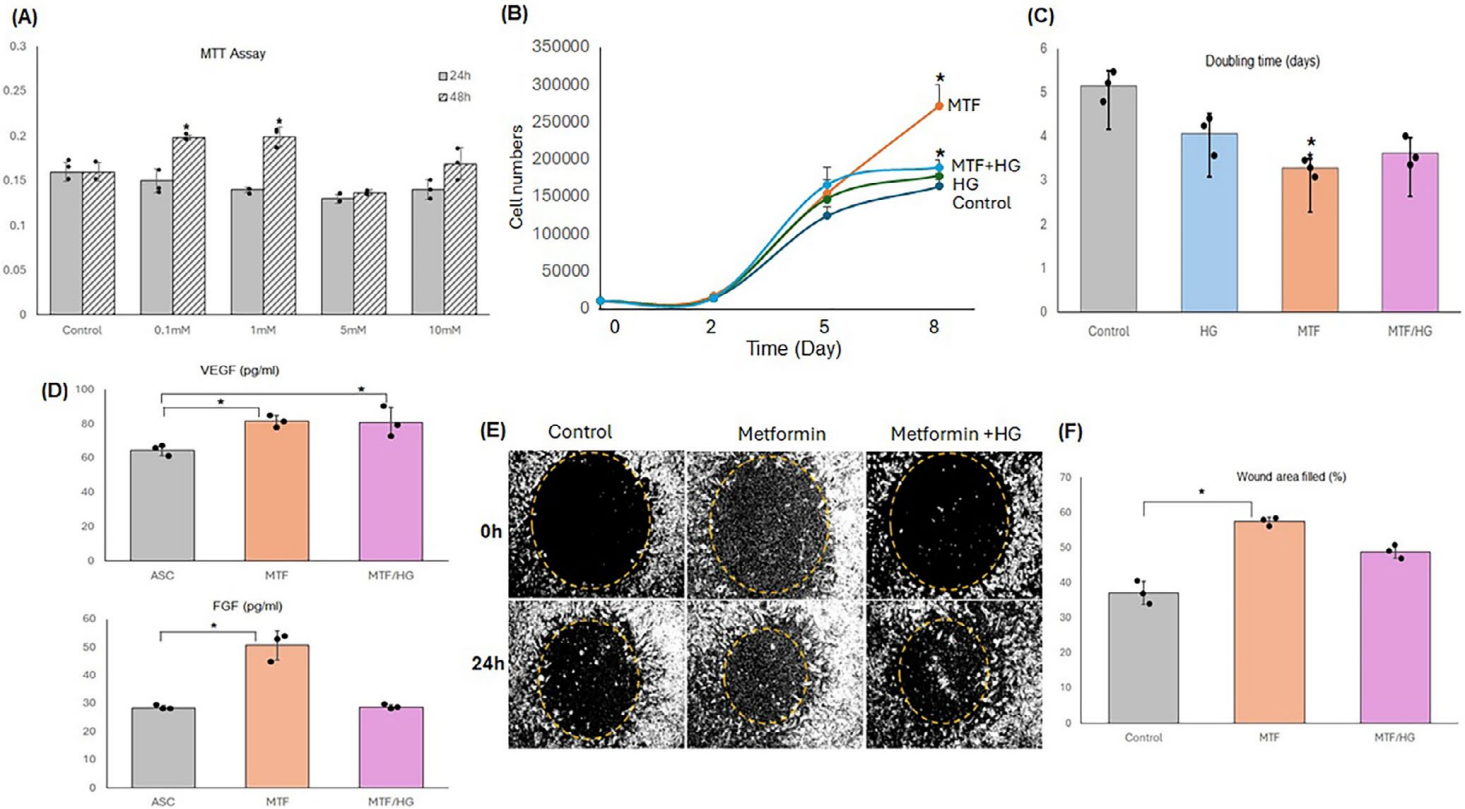
Metformin enhances ASC proliferation and paracrine angiogenic factor secretion. (A) MTT assay showing the effects of metformin (0–10 mM) on ASC metabolic activity at 24 and 48 h. (B) Longitudinal growth curves of ASCs cultured under control, metformin (MTF), high‐glucose (HG) and metformin plus high‐glucose (MTF + HG) conditions. (C) Quantification of population doubling time under the indicated treatment conditions. (D) ELISA quantification of VEGF and FGF secretion by ASCs under the indicated treatment conditions. (E) Representative images of human dermal fibroblast (HDF) scratch‐wound assays following co‐culture with ASCs under control, MTF and MTF + HG conditions at 0 and 24 h. Dashed lines indicate wound boundaries. (F) Quantification of HDF wound closure at 24 h. Data are presented as mean ± SD, with individual biological replicates shown as individual dots. *n* = 3 independent biological replicates per group. Statistical significance was determined using two‐way ANOVA followed by post hoc test for (A), appropriate test for (B) and one‐way ANOVA followed by post hoc test for (C), (D) and (F). *p* < 0.05 was considered statistically significant.

Longitudinal growth curve analysis showed enhanced expansion of MTF‐treated ASCs compared with control ASCs (Figure 2B). Consistent with these findings, population DT was significantly reduced in MTF‐treated ASCs compared with controls (3.3 ± 0.33 days vs. 5.2 ± 0.6 days, *p* < 0.05; Figure 2C). Under high‐glucose conditions, MTF‐treated ASCs also showed a trend towards reduced population DT compared with controls (3.7 ± 0.48 days vs. 5.2 ± 0.6 days, *p* = 0.08), suggesting partial preservation of proliferative capacity under metabolic stress.

To assess paracrine angiogenic signalling, ELISA was performed to quantify secreted growth factors. MTF‐treated ASCs exhibited significantly increased VEGF secretion (1.3‐fold, *p* < 0.05) and FGF secretion (1.8‐fold, *p* < 0.05) compared with untreated ASCs (Figure 2D). These findings indicate that MTF enhances the pro‐angiogenic secretory profile of ASCs, although this effect was attenuated under high‐glucose conditions.

To determine functional relevance, a co‐culture scratch‐wound assay using HDFs was performed. HDF wound closure was significantly enhanced in the presence of MTF‐treated ASCs compared with control ASCs (Figure 2E,F, *p* < 0.05). Under high‐glucose conditions, HDF wound closure showed an intermediate, non‐significant increase compared with controls (*p* > 0.05), suggesting partial attenuation of the pro‐migratory effect under metabolic stress.

Collectively, these findings demonstrate that MTF promotes ASC proliferation and enhances paracrine pro‐regenerative signalling in surrounding cells.

### 3.3 MTF Promotes Endothelial Differentiation and Angiogenic Function of ASCs

To determine whether MTF enhances endothelial differentiation of ASCs, cells were cultured with MTF and VEGF under the indicated conditions. Endothelial differentiation was assessed by CD31 expression, while VEGF expression was evaluated as a pro‐angiogenic marker.

Under normoglycemic conditions, combined MTF and VEGF treatment significantly increased CD31 mRNA expression compared with VEGF treatment alone (2.44 ± 0.17 vs. 1.55 ± 0.21, *p* < 0.05; Figure 3A). Representative IF images showed increased CD31 expression following combined MTF and VEGF treatment (Figure 3C). VEGF mRNA expression was also higher following combined MTF and VEGF treatment, whereas this response was attenuated under high‐glucose conditions (Figure 3B).

**FIGURE 3:**
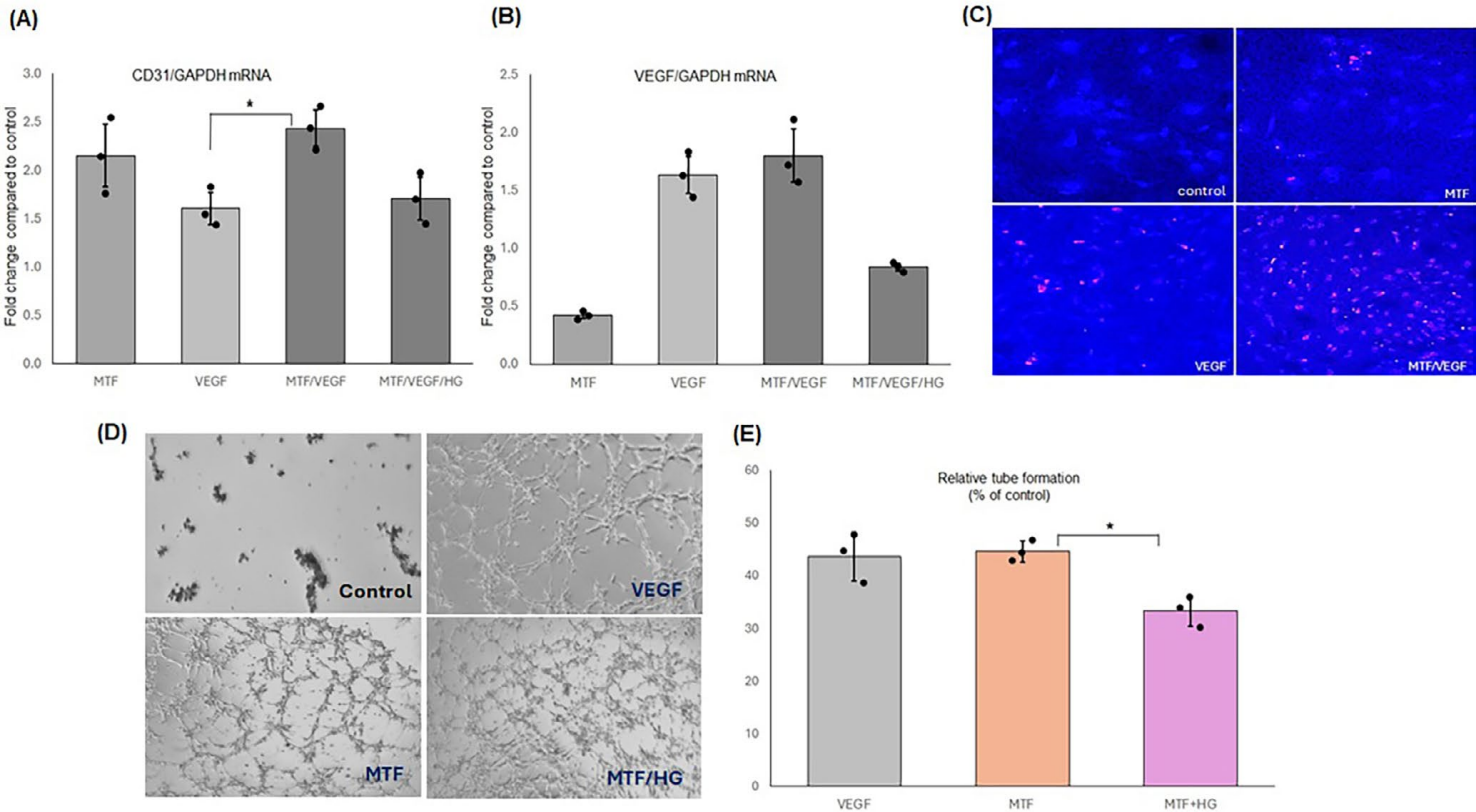
Metformin enhances VEGF‐associated endothelial differentiation and angiogenic function of ASCs. (A) Quantitative PCR analysis of CD31 mRNA expression in ASCs under the indicated treatment conditions following endothelial induction. (B) Quantitative PCR analysis of VEGF mRNA expression under the indicated treatment conditions. (C) Representative immunofluorescence images showing CD31 expression following endothelial induction. Nuclei were counterstained with DAPI. (D) Representative images of tube formation assays under the indicated treatment conditions. (E) Quantification of relative tube formation normalized to the untreated control group (100%). Data are presented as mean ± SD, with individual biological replicates shown as individual dots. *n* = 3 independent biological replicates per group. Statistical significance was determined using one‐way ANOVA followed by Tukey's multiple‐comparisons test. *p* < 0.05 was considered statistically significant.

Tube formation assays further demonstrated that high‐glucose exposure significantly reduced capillary‐like network formation by MTF‐treated ASCs compared with MTF‐treated ASCs under normoglycemic conditions (*p* < 0.05; Figure 3D,E).

Collectively, these findings demonstrate that MTF enhances VEGF‐associated endothelial differentiation and angiogenic activity of ASCs under normoglycemic conditions, whereas hyperglycaemia attenuates these effects. These findings are consistent with transcriptomic analyses identifying enrichment of angiogenesis‐related genes and signalling pathways following MTF treatment.

### 3.4 MTF Promotes ASC Adhesion and Endothelial Marker Expression on SIS Scaffolds

To evaluate the effect of MTF on ASC attachment to SIS scaffolds, ASC attachment was assessed by confocal microscopy. Confocal microscopy demonstrated ASC attachment to SIS membranes, with visually greater cell attachment following MTF treatment under both normoglycemic and high‐glucose conditions compared with untreated ASCs (Figure 4A).

**FIGURE 4:**
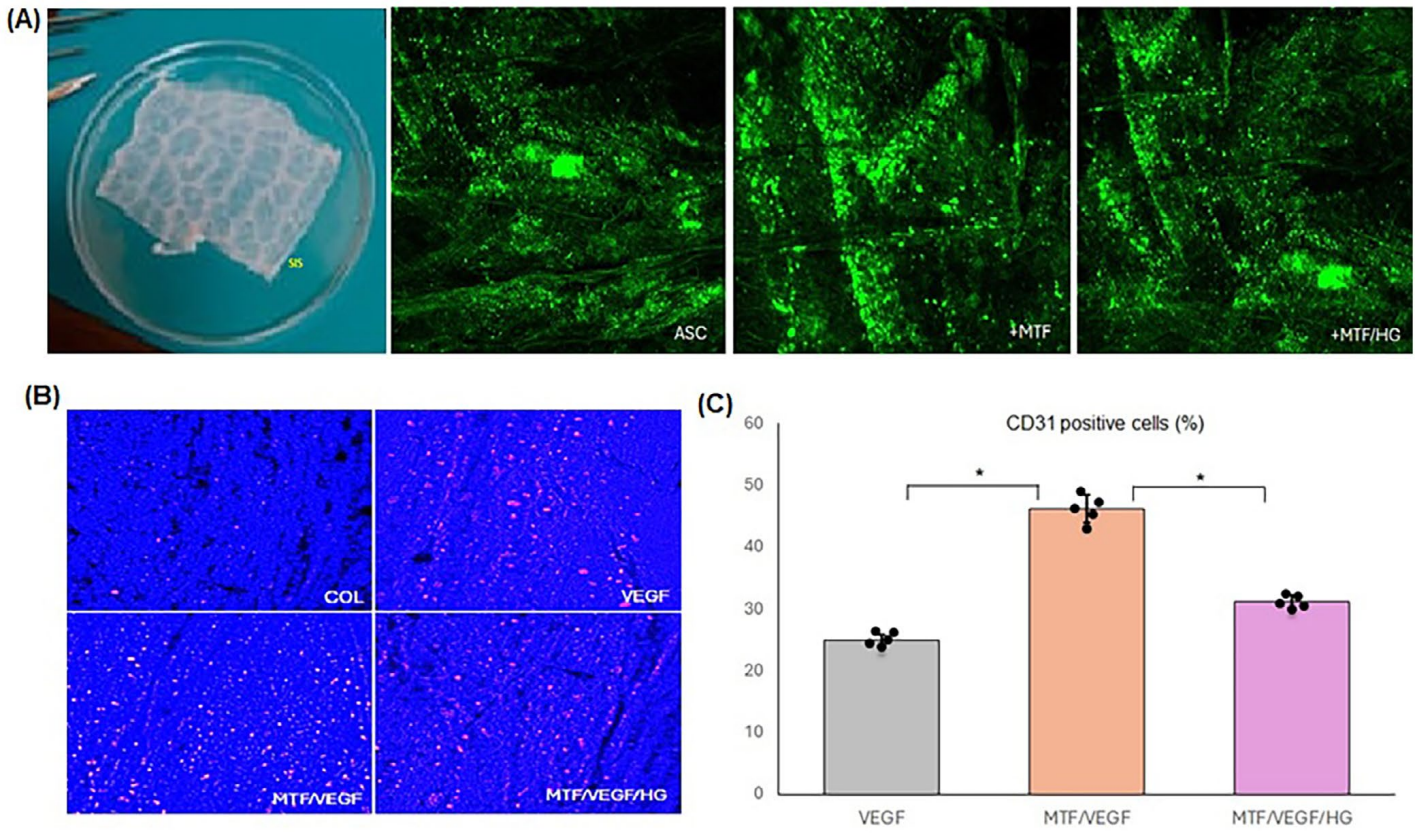
Metformin promotes endothelial marker expression by ASCs on SIS scaffolds. (A) Representative confocal microscopy images showing ASC attachment to small intestinal submucosa (SIS) scaffolds under the indicated treatment conditions. ASCs were labelled with CellTracker Green to visualize cell attachment. (B) Representative immunofluorescence images showing CD31 expression by ASCs cultured on SIS scaffolds under the indicated treatment conditions. Nuclei were counterstained with DAPI. (C) Quantification of CD31‐positive cells on SIS scaffolds after 10 days of culture under the indicated treatment conditions. Data are presented as mean ± SD, with individual biological replicates shown as individual dots. *n* = 5 independent biological replicates per group. Statistical significance was determined using one‐way ANOVA followed by Tukey's multiple‐comparisons test. *p* < 0.05.

To determine whether culture on SIS scaffolds influences endothelial marker expression, ASCs were seeded onto SIS scaffolds and maintained for 10 days in the presence of VEGF with or without MTF under normoglycemic and high‐glucose conditions. IF analysis demonstrated CD31 expression in ASCs cultured on SIS scaffolds (Figure 4B). Quantification revealed a significantly higher proportion of CD31‐positive cells following combined MTF and VEGF treatment compared with VEGF treatment alone (46.1% vs. 24.8%, respectively; Figure 4C; *p* < 0.05). Under high‐glucose conditions, the proportion of CD31‐positive cells in the MTF/VEGF group was significantly reduced compared with the corresponding normoglycemic condition (31.0% vs. 46.1%; Figure 4C; *p* < 0.05).

Collectively, these findings demonstrate that MTF promotes endothelial marker expression by ASCs within a 3D SIS scaffold environment and that this effect is attenuated under hyperglycemic conditions.

### 3.5 MTF Regulates Angiogenic and Inflammatory Gene Expression in ASCs

To further investigate the molecular mechanisms underlying the pro‐regenerative effects of MTF, we examined DEGs associated with angiogenesis and inflammatory signalling. Heatmap analysis revealed distinct expression patterns of angiogenesis‐related genes across treatment groups (Figure 5A). MTF treatment upregulated key genes involved in vascular development, endothelial function and tissue regeneration including representative angiogenic regulators, consistent with the enhanced endothelial differentiation observed in functional assays. In contrast, high‐glucose conditions altered the expression of multiple angiogenic factors, whereas MTF partially restored a pro‐angiogenic transcriptional profile in ASCs exposed to high glucose.

**FIGURE 5:**
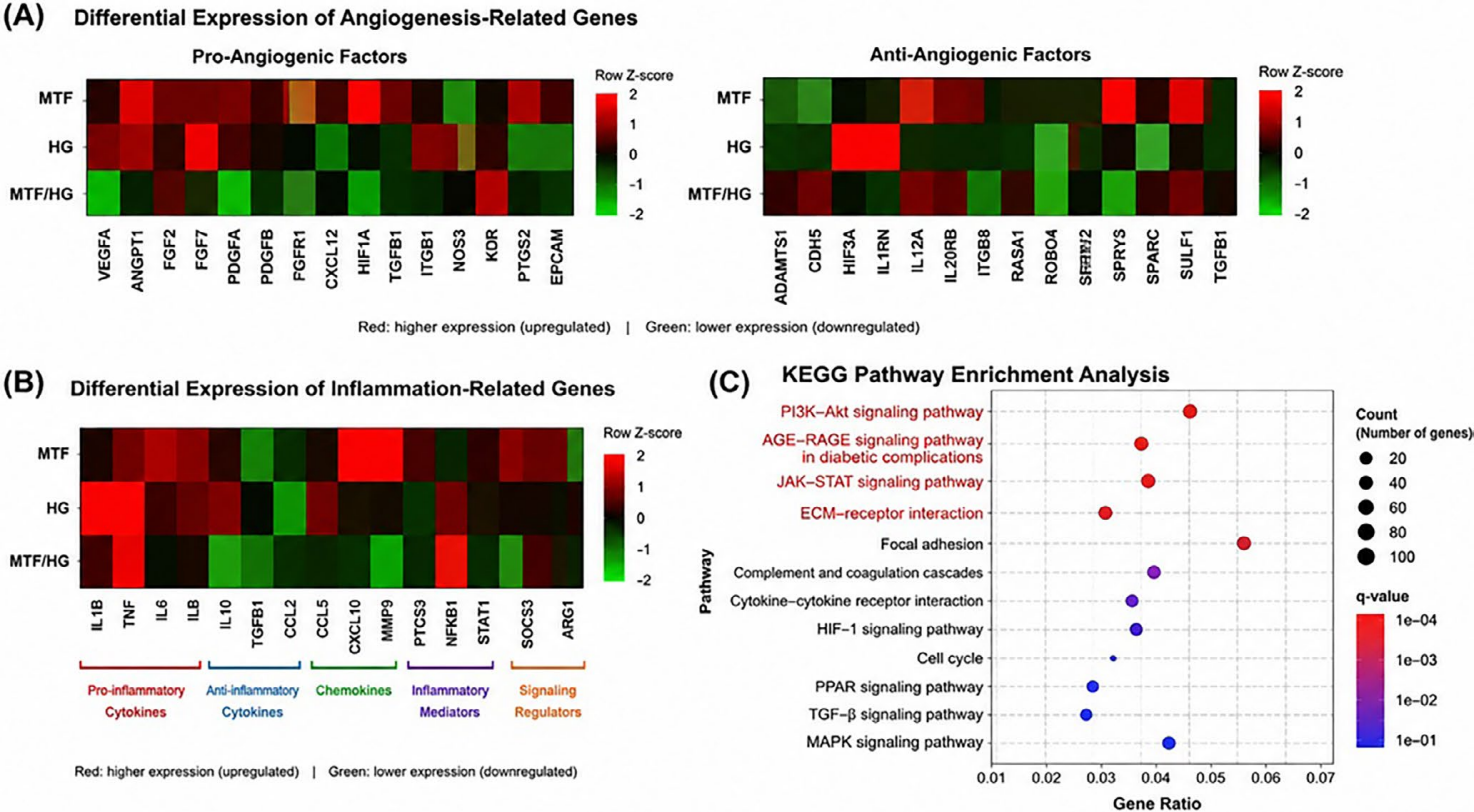
Metformin modulates angiogenic and inflammatory gene expression profiles in ASCs. (A) Heatmap showing RNA sequencing‐based expression patterns of angiogenesis‐associated differentially expressed genes (DEGs) among control, metformin‐treated (MTF), high‐glucose (HG) and metformin plus high‐glucose (MTF + HG) groups. (B) Heatmap showing expression profiles of inflammation‐associated DEGs across the treatment groups. (C) KEGG pathway enrichment analysis of DEGs identified in the indicated comparison. Bubble size represents the number of genes associated with each enriched pathway and colour indicates the adjusted *p* value. Gene expression levels are displayed as log_2_ fold change relative to control. RNA sequencing was performed using three biological replicates per group (*n* = 3). DEGs were identified using the predefined thresholds of |log_2_ fold change| ≥ 1.5 and adjusted *p* ≤ 0.05.

Analysis of inflammatory response genes demonstrated that MTF also modulated the expression of genes involved in cytokine signalling and immune regulation (Figure 5B). Compared with untreated controls, MTF‐treated ASCs exhibited altered expression of multiple inflammatory mediators, suggesting a shift towards a transcriptional programme favouring tissue repair.

To gain further insight into the biological processes associated with these transcriptional changes, KEGG pathway enrichment analysis was performed on angiogenesis‐ and inflammation‐associated DEGs (Figure 5C). Significantly enriched pathways included PI3K–AKT signalling (cell survival and proliferation), JAK–STAT signalling (cytokine‐mediated responses) and extracellular matrix organization pathways, all of which play central roles in regulating angiogenesis, inflammation and tissue remodelling.

Collectively, these findings demonstrate that MTF promotes a coordinated pro‐regenerative gene expression programme in ASCs through integrated modulation of angiogenic and inflammatory signalling networks.

## 4 Discussion

This study demonstrates that MTF enhances the functional properties of ASCs, supporting its potential application in regenerative medicine, particularly for impaired wound healing in diabetic environments. Collectively, our findings suggest that MTF modulates ASC proliferation, paracrine signalling, endothelial marker expression and scaffold integration, thereby promoting a more pro‐regenerative cellular phenotype.

MTF increased ASC proliferative capacity in a concentration‐dependent manner, with low doses promoting metabolic activity without cytotoxicity. Prolonged exposure further accelerated cell expansion, as reflected by reduced population DT. These findings are consistent with previous studies suggesting that MTF may regulate stem cell proliferation and metabolic activity through energy‐sensing pathways, including AMPK‐related signalling mechanisms [23, 30, 31, 32]. Importantly, while hyperglycaemic conditions partially attenuated this effect, MTF‐treated ASCs retained higher proliferative capacity than untreated controls, indicating a degree of resilience to metabolic stress. This may be, particularly, relevant to diabetic wound environments, where stem cell function is typically compromised.

In addition to proliferation, MTF enhanced the paracrine secretion of angiogenic factors, including VEGF and FGF. Previous studies have shown that MTF in combination with MSCs may enhance angiogenic responses through multiple mechanisms, including increased release of MSC‐derived EVs and upregulation of pro‐angiogenic mediators such as VEGF and stem cell factor (SCF), collectively supporting vascular differentiation [23, 33, 34]. Notably, our results demonstrated that VEGF secretion was maintained under both normoglycemic and hyperglycaemic conditions, whereas FGF secretion was partially reduced under high glucose. This differential response suggests that MTF may selectively preserve key angiogenic signalling components under metabolic stress. These findings are consistent with prior reports highlighting the importance of stem cell‐derived paracrine factors in neovascularization and tissue repair [14, 24, 35, 36]. Overall, these results suggest that MTF supports a pro‐regenerative ASC secretome characterized by sustained VEGF signalling and partially preserved growth factor production, which may contribute to vascularization and tissue repair in diabetic wound environments.

Fibroblast migration is a critical step in wound healing and impaired fibroblast activity is a hallmark of chronic diabetic wounds [37, 38, 39]. In our co‐culture system, MTF‐treated ASCs significantly enhanced fibroblast migration, potentially through paracrine signalling mechanisms. Although this effect was partially attenuated under hyperglycaemic conditions, an overall pro‐migratory response was maintained, suggesting that MTF‐treated ASCs retain the capacity to support fibroblast function under metabolic stress. These findings support the concept that ASC‐mediated intercellular communication contributes to tissue remodelling and wound repair, even in compromised diabetic‐like environments [40].

ASCs have the capacity to acquire endothelial‐like characteristics in response to pro‐angiogenic cues, potentially contributing to neovascularization and tissue repair [41, 42, 43]. MTF also promoted endothelial marker expression in ASCs, particularly in combination with VEGF, as evidenced by increased CD31 expression and enhanced tube formation under normoglycemic conditions. However, this synergistic effect was attenuated under high‐glucose conditions, consistent with previous studies reporting impaired angiogenic responses in diabetic microenvironments [44, 45, 46]. These findings suggest that metabolic stress remains a key limiting factor in endothelial‐associated responses and angiogenic function, potentially reducing the regenerative capacity of ASCs in diabetic wound environments. Collectively, our results support the use of MTF as an adjunct to enhance ASC‐mediated vascular regeneration while highlighting the importance of metabolic dysfunction when developing regenerative therapies for diabetic wounds.

Preclinical and early clinical studies have demonstrated that combining MTF with biomaterials such as hydrogels and scaffolds can enhance therapeutic outcomes in regenerative applications [47, 48]. Consistent with these observations, MTF significantly improved ASC adhesion to clinically relevant SIS scaffolds in our study, a critical factor for cell retention, survival and tissue integration following implantation. Enhanced ASC adhesion was observed under both normoglycemic and hyperglycaemic conditions, suggesting that MTF may facilitate cell–scaffold interactions even in metabolically compromised environments. Although the underlying mechanisms were not directly investigated, previous studies have reported that MTF can influence cytoskeletal remodelling and integrin expression, processes that are closely associated with cell attachment and biomaterial interactions [49, 50]. Importantly, ASCs cultured on SIS scaffolds maintained their angiogenic potential, supporting the translational relevance of combining metabolic preconditioning with biomaterial‐based delivery strategies. Together, these findings suggest that MTF may enhance both cell–scaffold interactions and the regenerative functionality of ASCs, providing a promising approach for tissue engineering and chronic wound repair.

Transcriptomic analysis further identified molecular pathways associated with the therapeutic response to MTF, including pathways related to angiogenesis, inflammation, cell survival and extracellular matrix remodelling, such as PI3K–AKT, JAK–STAT and AGE–RAGE signalling. These molecular findings provide additional context for the functional effects observed in this study and suggest that MTF may influence multiple biological processes relevant to tissue repair. Importantly, the transcriptional response varied according to glucose conditions, highlighting the influence of metabolic stress on regenerative responses.

Despite these promising findings, several limitations should be acknowledged. First, these in vitro studies do not fully recapitulate the complexity of the wound‐healing environment in vivo. Second, hyperglycaemic culture conditions model only one aspect of diabetic pathology and do not capture the chronic inflammatory, vascular and systemic metabolic abnormalities associated with diabetes. Finally, although transcriptomic analysis identified several signalling pathways potentially associated with the observed functional effects, direct mechanistic validation was not performed. Future studies using diabetic wound‐healing models and targeted pathway analyses will be necessary to confirm and extend these findings.

In summary, MTF enhances ASC proliferation, paracrine angiogenic signalling, endothelial marker expression and scaffold adhesion, collectively supporting a pro‐regenerative phenotype. However, these beneficial effects are partially attenuated under hyperglycaemic conditions, underscoring the importance of metabolic context in regenerative therapies. By integrating functional, biomaterial and transcriptomic analyses, this study provides evidence that MTF preconditioning may represent a promising strategy to improve ASC‐based approaches for wound healing and tissue regeneration.

## Author Contributions

**Kristina Lee Khaw:** investigation, data curation, methodology, formal analysis, conceptualization, writing – original draft. **Max von Suskil:** investigation, data curation, methodology, formal analysis, writing – original draft. **Andrew Bohner:** investigation, data curation, methodology. **Russell J. Buono:** conceptualization, data curation, validation, writing – review and editing. **Steven C. Bonawitz:** investigation, resources. **Kathryn Behling:** investigation, resources. **Michael E. DiSanto:** conceptualization, investigation, resources, writing – review and editing. **Ping Zhang:** conceptualization, data curation, formal analysis, methodology, validation, writing – original draft, writing – review and editing, funding acquisition.

## Funding

This work was supported by the New Jersey Health Foundation (PC‐213‐24) 2024.

## Conflicts of Interest

The authors declare no conflicts of interest.

## Data Availability

The data that support the findings of this study are available from the corresponding author upon reasonable request.
